# Neck and Shoulder Morbidity in Patients with Oral Cancer and Clinically Negative Node Neck Status: A Comparison between the Elective Neck Dissection and Sentinel Lymph Node Biopsy Strategies

**DOI:** 10.3390/healthcare10122555

**Published:** 2022-12-16

**Authors:** Gerben van Hinte, Koen P. A. Withagen, Remco de Bree, Caroline M. Speksnijder

**Affiliations:** 1 Department of Rehabilitation, Radboud University Medical Center, 6525 GA Nijmegen, The Netherlands; 2 Department of Head and Neck Surgical Oncology, University Medical Center Utrecht, University of Utrecht, 3584 CX Utrecht, The Netherlands; 3 Department of Oral and Maxillofacial Surgery and Special Dental Care, University Medical Center Utrecht, University of Utrecht, 3584 CX Utrecht, The Netherlands

**Keywords:** head and neck cancer, lymph nodes, quality of life, sentinel lymph node biopsy, elective neck dissection, neck and shoulder morbidity

## Abstract

The choice for the most optimal strategy for patients with a cT_1-2_N_0_ carcinoma of the oral cavity, sentinel lymph node biopsy (SLNB) or elective neck dissection (END), is still open for debate in many head and neck cancer (HNC) treatment centers. One of the possible benefits of the less invasive SLNB could be reduced neck and shoulder morbidity. Recent studies have shown a benefit in favor of SLNB the first year after intervention, but the long-term consequences and differences in neck morbidity remain unclear. This cross-sectional study aimed to research differences in neck and shoulder morbidity and Health-Related Quality of Life (HR-QoL) in patients with a cT_1-2_N_0_ carcinoma of the oral cavity, treated with either END or SLNB. Neck and shoulder morbidity and HR-QOL were measured with patient-reported questionnaires (SDQ, SPADI, NDI, NDII, EORTC-QLQ-C30, EORTC-QLQ-HN35) and active range of motion (AROM) measurements. In total 18 patients with END and 20 patients with SLNB were included. We found no differences between END and SLNB for long-term neck morbidity, shoulder morbidity, and HR-QOL. The significant differences found in the rotation of the neck are small and not clinically relevant.

## 1. Introduction

Head and neck squamous cell carcinomas arise in the mucosal linings of the upper respiratory digestive tracts and are considered an important part of the global burden of cancer [1]. The most frequent anatomical site for head and neck squamous cell carcinomas is the oral cavity. Approximately half of the patients are diagnosed with early stage cT_1-2_ (<4 cm without cancer cells present in nearby structures, lymph nodes or distant sites) oral cavity squamous cell carcinoma (OCSCC) [2]. Advancements in medical treatment have improved survival, but a high number of patients experience treatment-related morbidity regarding physical, social, emotional, and psychological health [3,4]. Patients can experience limitations in functions of the head and neck, activities of daily living, and oral functioning [5,6,7,8,9]. Limitations in physical health can remain present in the long term and are strongly correlated with a lower health-related quality of life (HR-QoL) [3,10]. Limitations in active range of motion (AROM) of neck and shoulder are highly prevalent in patients with OCSCC [5,11].

The cT_1-2_ OCSCC patient is labeled as a clinically negative neck (cN_0_) when during the pretreatment phase no regional lymph nodes metastases are detected by palpation, fine needle aspiration cytology, and/or imaging techniques. The cN_0_ patients are, however, still at risk of occult lymph node metastases that are present in 20 to 30% of cN0 OSCC patients [12]. The two most used management strategies to improve locoregional recurrence-free survival as compared to observation are the elective neck dissection (END) and the sentinel lymph node biopsy (SLNB). The END usually consists of the removal of all lymph nodes at risk located at levels I, II, III, and sometimes level IV [13]. The SLNB consist of a surgical procedure in which the first draining lymph node(s), called sentinel lymph nodes (SLNs), are identified, removed, and examined by a pathologist to determine whether cancer cells are present. Only if positive nodes are present, a neck dissection is subsequently performed. Thereby, SLNB limits possible surgical overtreatment of the neck up to 80% compared to a strategy in which all cN_0_ patients undergo END. Recent research has shown that the END and SLNB strategies have comparable outcomes on neck node recurrence-free survival at 2 years follow up (89.6% for END and 90.7% for SLNB) [14]. With the SLNB being considered a less invasive strategy compared to END it is expected to cause less local morbidity and better HR-QoL in comparison to END and therefore less need for physical therapy intervention or screening [15,16]. However, the choice for the most optimal strategy for the cN_0_ patients is still open for debate in many head and neck cancer (HNC) treatment centers. It is also unclear if a patient undergoing SLNB should also be screened for neck and shoulder limitations by a physical therapist as is advised for patient undergoing END in treatment guidelines in The Netherlands [17]. It is therefore important to research differences in neck and shoulder morbidity and health-related quality of life (HR-QoL). Previous research reported lower postoperative shoulder morbidity for SLNB compared to END [14,18,19,20]. A patient’s perspective study showed that patients undergoing SLNB preferred this strategy over END [20]. However, in two studies the END group received END after SLNB [18,20] and four studies lack objective measurements on neck and shoulder morbidity [14,18,19,21]. Our previous longitudinal comparative study showed less shoulder morbidity at 6 weeks post intervention for SLNB but lacks information on neck morbidity and long-term follow-up (>1 year). Although less invasive, a recent systematic review showed that the difference in HR-QoL between END and SLNB remains unclear [22]. Therefore, more insight is needed into the difference between END and SLNB regarding long-term neck and shoulder morbidity and HR-QoL. This study aimed to compare shoulder and neck morbidity and HR-QoL between cT_1-2_N_0_ OCSCC patients with cN_0_ undergoing END or SLNB. For OCSCC patients with a clinically negative neck, we hypothesized less neck and shoulder morbidity and better HR-QoL after SLNB as compared to the END strategy.

## 2. Materials and Methods

### 2.1. Study Setting and Participants

This cross-sectional study included patients who were treated for cT_1-2_N_0_ OCSCC between 2012 and 2019 at the UMC Utrecht, The Netherlands. The study was conducted according to the principles of the Declaration of Helsinki 2013 and in accordance with the Medical Research Involving Humans Subjects Act (WMO). The research protocol was approved by the Ethics Committees of UMC Utrecht (NL68148.041.18). Informed consent was obtained from all participating patients.

Patients were included if they: (1) had cT_1-2_N_0_ OCSCC, (2) underwent END or SLNB, and (3) were at least 18 years old. Patients were excluded when they: (1) received postoperative radiotherapy, (2) had recurrent OCSCC, (3) were unable to read Dutch and/or complete the questionnaires, (4) had a history of neck or shoulder surgery, (5) underwent END after positive SLNB, and (6) were treated with a bilateral neck dissection. This study followed guidelines provided by the Strengthening The Reporting of OBservational Studies in Epidemiology (STROBE) statement [23].

### 2.2. Study Procedure

Patients scheduled for usual care follow-up appointments at the UMC Utrecht were informed and asked to participate in the study. Informed consent was obtained before the measurements. Demographic, participant, and treatment characteristics were collected from the electronic hospital treatment and registration system: age, gender, END or SLNB strategy, tumor location (maxilla, mandibular, floor/mouth, cheek), treated side (left/right), time since treatment and cTNM-stage (cT_1-2_). During the research appointment additional patient characteristics were collected for height (meters) weight (kilograms), alcohol consumption after treatment (units of alcohol per day), tobacco use (pack-years), use of physiotherapy (yes/no), number of physiotherapy treatments in the past years and if they received physiotherapy for head and neck related problems (yes/no). Data was collected between February–December 2019.

#### 2.2.1. Shoulder Morbidity

The Shoulder Disability Questionnaire (SDQ) is a valid and reliable pain-related disability questionnaire, which contains 16 items describing common situations that may induce symptoms in patients with shoulder disorders and was the primary outcome of this study [24]. All items refer to the preceding 24 h.

The Shoulder Pain and Disability Index (SPADI) is a valid and reliable self-report questionnaire that measures shoulder pain and disability experienced during the last week [25].

#### 2.2.2. Neck Morbidity

The Neck Disability Index (NDI) is a valid and reliable self-assessment questionnaire that measures neck disability such as pain and headache experienced by patients during the last four weeks [26].

#### 2.2.3. Shoulder and Neck Morbidity

The Neck Dissection Impairment Index (NDII) is a valid and reliable self-rated questionnaire that assesses both neck and shoulder morbidity in patients with a neck dissection [27,28].

#### 2.2.4. Shoulder and Neck Active Range of Motion

The AROM for the shoulder was measured for external rotation, abduction, and forward flexion. The AROM of the neck was measured for rotation, flexion, extension, and lateral flexion. Objective measurements on active range of motion (AROM) of the neck was performed with the participant in a sitting position and in a standing position for shoulder AROM according to a predefined measurement protocol For both shoulder and neck AROM, the side of treatment was considered the ipsilateral AROM and the opposite side the contralateral AROM [29]. AROM was measured using the MicroFET 6 electronic inclinometer (Hoggan Health Industries; West Jordan, UT, USA) ^®^. For AROM in the transverse plane (external rotation of the shoulder) the universal goniometer was used [30].

#### 2.2.5. Quality of Life

The European Organization for Research and Treatment for Cancer—Quality of Life Questionnaire—Core 30 questions (EORTC-QLQ-C30) is a valid and reliable self-report questionnaire that assesses the multiple dimensions of Quality of Life among cancer patients [31].

The European Organization for Research and Treatment for Cancer-Quality of Life Questionnaire—Head and Neck 35 questions (EORTC-QLQ-HN35) is an HNC-specific Quality of Life Questionnaire [32].

### 2.3. Sample Size

We used a convenience sample for this comparative study. This limits the power to detect true differences between groups giving the study are more explorative design.

### 2.4. Statistical Analysis

Descriptive statistics were used to describe patient characteristics. Categorical outcomes are presented as numbers and percentages. Normal distributed continuous outcomes are presented as mean and standard deviation and skewed continuous outcomes, ordinal outcomes as median and interquartile range. Normal distribution was tested with the Shapiro–Wilk Test of Normality and equality of variances with Levene’s test. Normally distributed continuous data were analyzed by the independent t-test. The Mann–Whitney U test was used for both ordinal and skewed continuous data and the Chi-Square test for categorical data. Means and standard deviations and medians and interquartile ranges were presented for all outcome measurements. Possible confounders were selected based on known effects on shoulder morbidity, neck morbidity, and HR-QoL (age, time since treatment) [5]. The influence of the confounders on the outcome measurement was tested through association analysis using the Pearson’s (continuous data) and Spearman’s rank correlation coefficients (ordinal and non-normal distributed continuous data). The level of statistical significance was set at *p* < 0.05. Statistical analyses were performed using SPSS IBM version 25.0 (IBM Corporation, Armonk, NY, USA).

## 3. Results

In total, 38 patients agreed to participate in this study, after informed consent all patients completed the measurements. Demographic and clinical characteristics are described in Table 1. Of all included patients, eighteen patients underwent END (47.4%) and twenty patients SLNB (52.6%). The participants in the END group were older (*p* = 0.001), were measured at a longer time since treatment (*p* < 0.000), varied more in tumor location (*p* = 0.032), and more frequently consulted a physical therapist (*p* = 0.024), also indicated by a higher number of physical therapy treatments received (*p* = 0.022). The six END patients that were treated by a physical therapist received between two and more than a hundred treatment sessions in comparison to one SLNB patient that had six physiotherapy treatment sessions. Shoulder and neck related problems were the indication for physical therapy treatment of all patients (100%).

**Figure 1:**
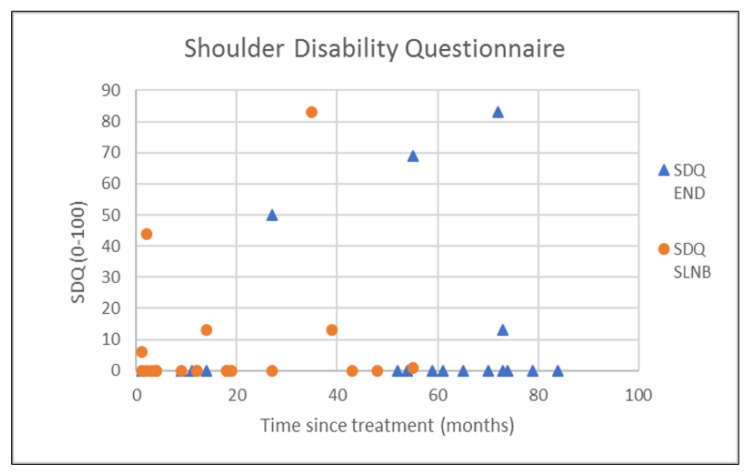
Shoulder disability questionnaire (SDQ) in relation to time since treatment.

**Figure 2:**
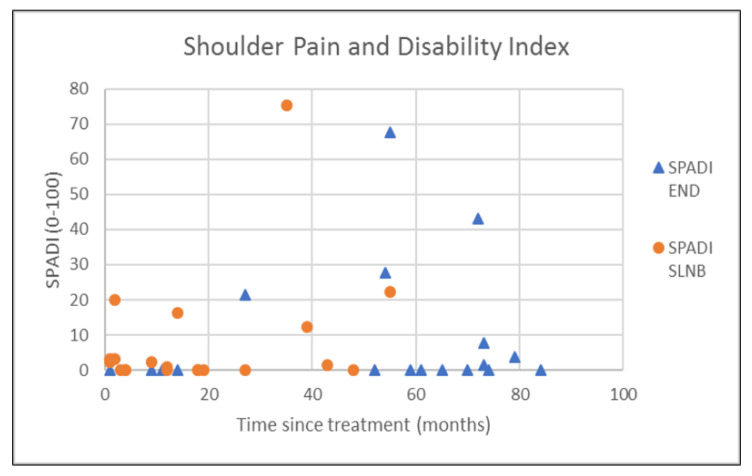
Shoulder pain and disability index (SPADI) in relation to time since treatment.

**Figure 3:**
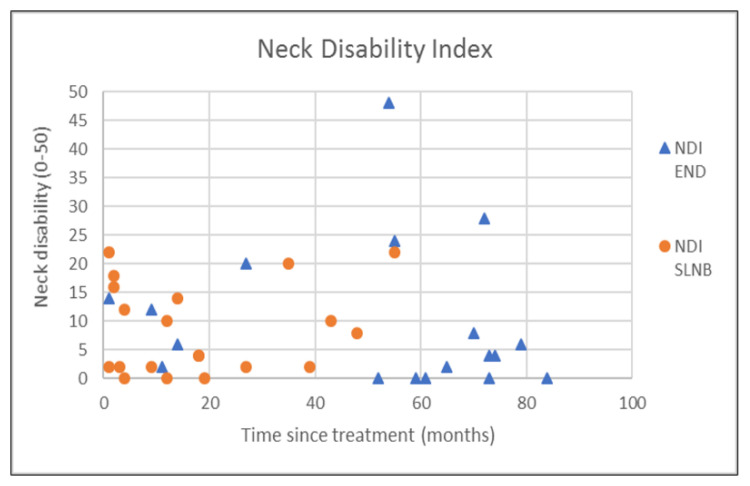
Neck disability index (NDI) in relation to time since treatment.

**Figure 4:**
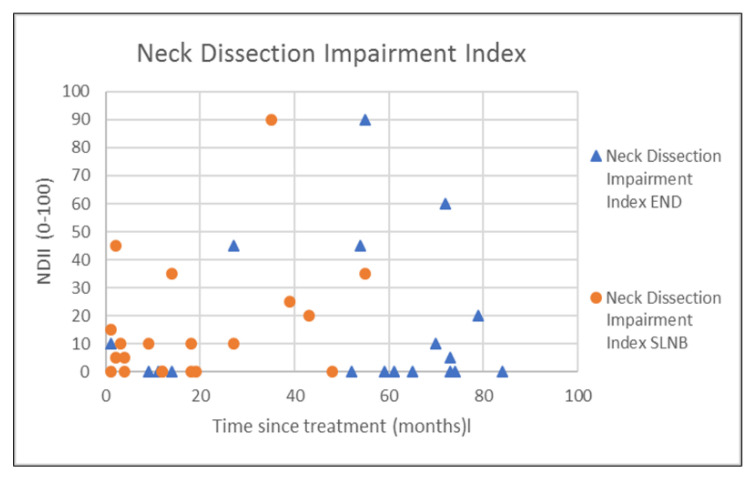
Neck dissection impairment index (NDII) in relation to time since treatment.

**Figure 5:**
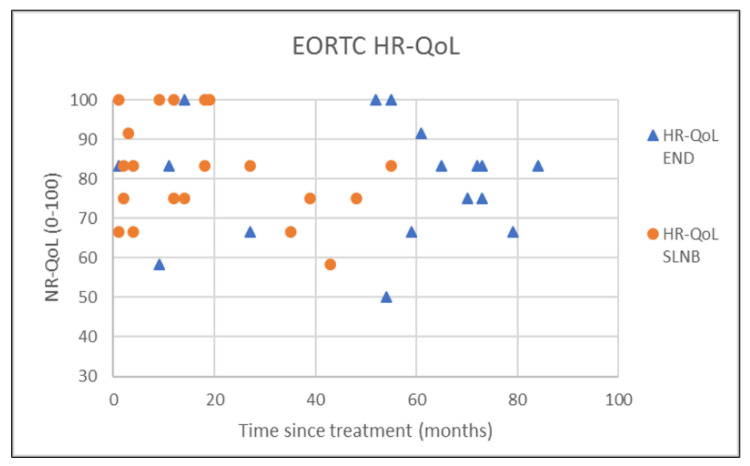
EORTC HR-QoL C30 in relation to time since treatment.

**Figure 6:**
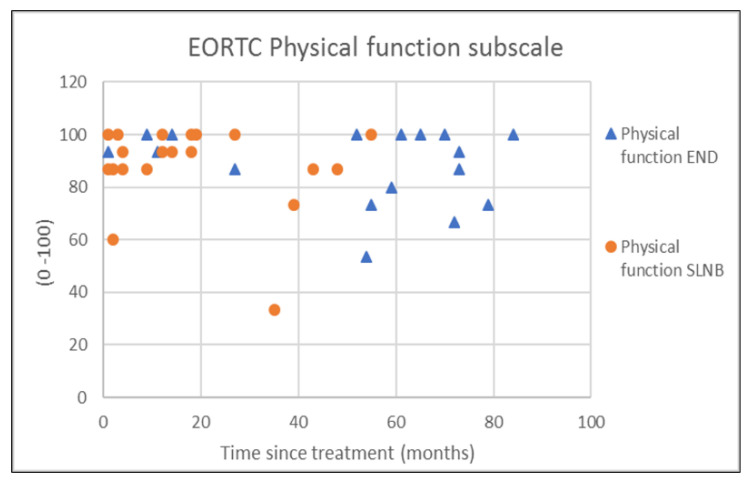
EORTC HR-QoL C30 Physical function subscale in relation to time since treatment.

**Figure 7:**
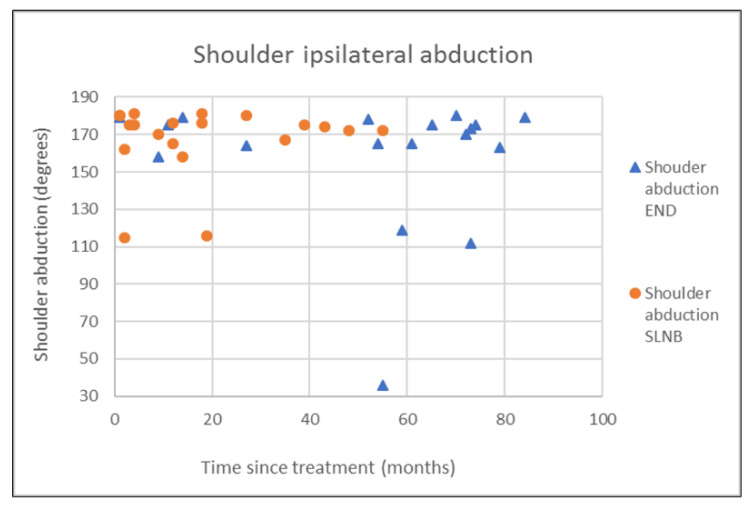
Shoulder ipsilateral abduction in relation to time since treatment.

**Figure 8:**
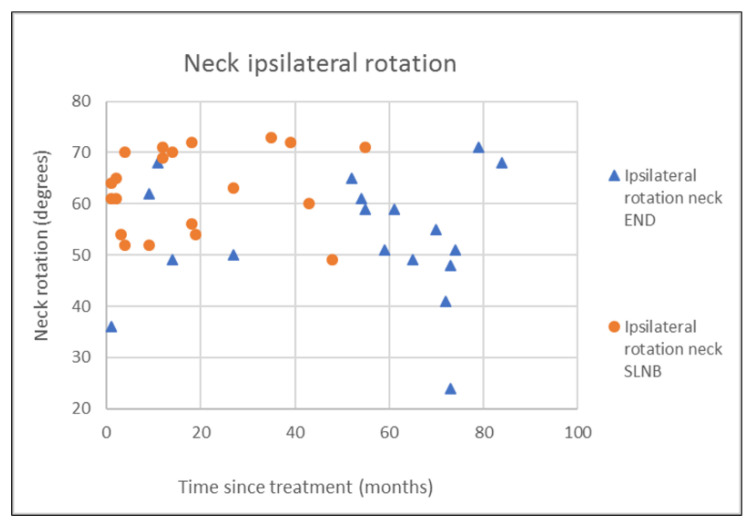
Neck ipsilateral rotation in relation to time since treatment.

**Figure 9:**
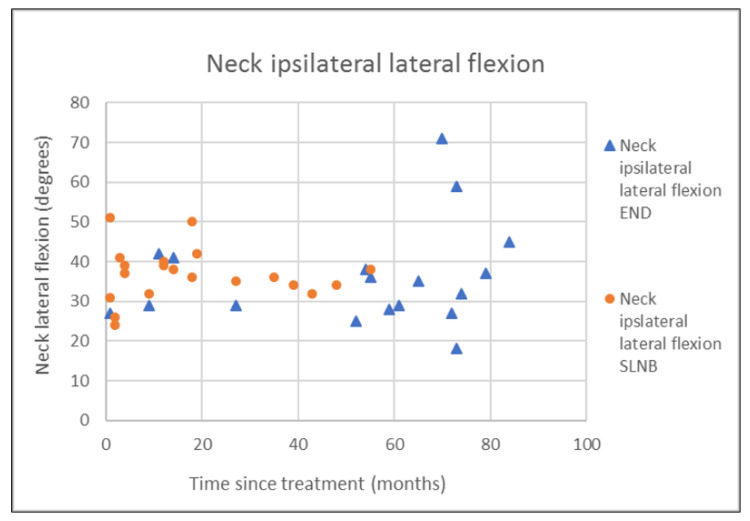
Neck ipsilateral lateral flexion in relation to time since treatment.

The outcomes of questionnaires on neck and shoulder morbidity, HR-QoL showed no significant differences between the End and the SLNB group (Table 2). For AROM measurements there were significantly better scores for ipsilateral (*p* = 0.008) and contralateral rotation (*p* = 0.029) of the neck for the SLNB strategy compared to END. No association was demonstrated between the possible confounding variables age and time since treatment and the outcome measurements. Because time since treatment was significantly different between END and SLNB at baseline, we chose to visually represent the relationship between time since treatment and the nine clinically most relevant outcome measurements in Figure 1, Figure 2, Figure 3, Figure 4, Figure 5, Figure 6, Figure 7, Figure 8 and Figure 9.

## 4. Discussion

This study found no differences in shoulder morbidity, neck morbidity, and health-related quality of life between the END and the SNLB treatment strategy for patients with clinically negative neck T1/T2 carcinoma of the oral cavity. The differences found between strategies for ipsilateral and contralateral rotation of the neck are significant but small, and within known measurement errors limiting the clinical relevance [33]. In addition, the median AROMs for shoulder and neck, for both strategies are above age and gender stratified reference values, indicating no limitations in shoulder and neck function for both strategies [33]. The absence of differences between strategies is not in line with our hypothesis that patients undergoing the SLNB strategy would experience less treatment-related morbidity due to the less invasive procedure. END patients reported more frequently that they had consulted a physical therapist for shoulder and neck-related problems. END patients also reported slightly higher levels of shoulder and neck morbidity. This higher consumption of outpatient physical therapy could be related to the standard clinical physical therapy consultation of END patients in our clinic. Because it was thought that SLNB patients do not generally experience substantial shoulder and neck problems, consultation and information by a physiotherapist were not routinely provided to SLNB patients. This has led to more outpatient physical therapy referrals of END patients in comparison to SLNB patients. Although not statistically significant, the END patients showed higher shoulder- and neck morbidity (SDQ, SPADI, NDII). The visual inspection of Figure 1 shows that 2 participants in the SLNB group and 3 patients in the END group have a shoulder function of below 120 degrees or shoulder abduction. A decrease in shoulder abduction is one of the clinical indicating accessory nerve palsy [34]. This finding supports the importance of awareness and screening both END and SLNB patients on shoulder morbidity during regular follow-up consultations. Two other cross-sectional studies have researched the difference in shoulder morbidity at equally long moments of follow-up (1.9 to 6.0 years) and both studies showed better outcomes for SLNB compared to END [18,19]. The study by Govers et al. had a larger sample size (*n* = 181) which makes it more sensitive to detect differences between groups [19]. The other study by Murer et al. [18] measured very low incidence scores in neck and shoulder morbidity compared to other studies, possibly limiting the clinical relevance of the reported differences. A third study that longitudinally researched shoulder morbidity found worse scores for shoulder morbidity at 6 months for patients undergoing END (within-group comparison with baseline), but no differences were found when comparing both strategies over time [21]. A randomized prospective study by Garrel et al. demonstrated that with the use of a self-reported questionnaire, shoulder morbidity was significantly lower at 2, 4, 6, and 12 months in favor of the SLNB strategy, but not at month 24 [14]. Although this study is lacking a baseline measurement and specific AROM measurements, it confirms our findings that treatment-related morbidity at longer follow-up (>12 months) is less prevalent and outcomes in shoulder morbidity are comparable between the two strategies at long-term follow-up. We found no differences in cancer generic or head and neck-specific HR-QoL. This is in contrast with the previous research that found better health utility scores (EQ-5D-3L) for SNLB compared to END representing HR-QoL. The study by Flach et al. also compared HR-QoL (EORTC-QLQ-C30, EORTC-QLQ-H-N35) and also found no significant difference between the two strategies [21]. In our study, 33% of the END patient received post-operative outpatient physiotherapy in comparison to only 5% of SLNB patients. In total five out of six END patients received more than 35 physiotherapy treatments, indicating persistent shoulder- and neck morbidity. This finding is in line with the findings of the study by Garrel et al. where significantly more physical therapy treatment was reported by the END patients. It is unclear if this could also be related to standardized clinical physiotherapy consultations and referral. [14]. Referral to a physical therapist has to be considered when pain or limitations in the shoulder or neck AROM are present.

Our study was the first to measure differences in shoulder and neck morbidity, and HRQoL between the END and SLNB strategy, with a set of validated patient-reported questionnaires and physical range of motion measurements. The patients included in our END strategy group are not derived indirectly from the SLNB group (after a positive lymph node) as in other studies, and therefore give a more valid representation [21]. Important factors to take into account when evaluating our findings are the cross-sectional design, the small sample size, and a median moment of measurement of 60 months for the END group and 13 months for the SLNB. The END and SLNB groups both had a median time since treatment that can be labeled as a long term moment of follow-up, where contrast is expected to be smaller due to natural recovery over time. The small sample sizes limit the power to identify true differences in neck and shoulder morbidity. With low incidence of neck and shoulder morbidity and relatively large standard deviations for the SDQ questionnaire, this would require larger groups (>100 participants). This limits the generalizability of our results and gives the findings a more explorative character. Future research could be focused on the longitudinal course of shoulder and neck morbidity and HR-QoL for both the END and SLNB treatment strategies. It would be specifically of interest to have multiple measurements during the initial post-intervention phase because it is expected that possible benefits from the less invasive SLNB strategy are to be found in the first 6 months. When further research would confirm that patients undergoing SLNB can also experience shoulder and neck morbidity, treatment guidelines and information that is given to patients who have undergone an SLNB should be updated.

## 5. Conclusions

We found no differences between the END and SLNB treatment strategies regarding shoulder morbidity, neck morbidity, and HR-QOL as measured with patient-reported questionnaires after long-term follow-up. The significant differences between strategies found in forward flexion of the shoulder and lateral flexion of the neck are small and not clinically relevant.

## Figures and Tables

**Table 1:** Demographic and clinical characteristics.

	END (N = 18)	SLNB (N = 20)	*p*-Value
Sex (*n*, %)			0.299 ˠ
Male	12 (66.7)	10 (50.0)	
Female	6 (33.3)	10 (50.0)	
Age in years (median, IQR)	72.0 (11.0)	63.5 (16.5)	0.001 †**
BMI (median, IQR)	24.8 (6.4)	26.4 (8.0)	0.907 °
Time since treatment in months (median, IQR)	60.0 (49.3)	13.0 (29.8)	0.000 †***
Treated side (*n*, %)			0.107 ˠ
Left	11 (61.1)	7 (35.0)	
Right	7 (38.9)	13 (65.0)	
TNM-stage (*n*, %)			0.552 ˠ
cT1	10 (55.6)	13 (65.0)	
cT2	8 (44.4)	7 (35.0)	
Tumor location (*n*, %)			0.032 ˠ*
Mandible	4 (22.0)	2 (10.0)	
Tongue/flour of mouth	10 (55.6)	18 (90.0)	
Buccal mucosa	4 (22.0)	0 (0.0)	
Pack years (median, IQR)	6.7 (38.8)	1.1 (37.5)	0.613 †
Alcohol use in units daily (median, IQR)	1.0 (1.6)	0.0 (1.6)	0.346 †
Post-operative physiotherapy (*n*, %)			0.024 ˠ*
Yes	6 (33.3)	1 (5.0)	
No	12 (66.7)	19 (95.0)	
Numbers of physiotherapy treatment (median, IQR)	0.0 (27.5)	0.0 (0.0)	0.022 †*

*: *p* < 0.05; **: *p* < 0.01; ***: *p* < 0.001; ˠ: chi-square test; †: Mann–Whitney U test; °: independent *t*-test; BMI: body mass index; END: elective neck dissection; IQR: interquartile range; SLNB: sentinel lymph node biopsy.

**Table 2:** Morbidity and active range of motion of neck and shoulder and health related quality of life.

	END (N = 18)		SLNB (N = 20)		
	Mean (SD)	Median (IQR)	Mean (SD)	Median (IQR)	*p*-Value
SDQ	11.9 (26.3)	0.0 (3.25)	8.0 (20.4)	0.0 (4.75)	0.828 †
SPADI	9.6 (18.9)	0.0 (11.2)	8.0 (17.4)	1.2 (10.0)	0.635 †
NDI	9.9 (12.9)	5.0 (15.5)	8.5 (7.8)	6.0 (13.5)	0.825 †
NDII	7.9 (13.2)	0.0 (13.1)	7.9 (11.1)	5.0 (11.9)	0.442 †
AROM shoulder					
External rotation ipsi	62.1 (14.8)	64.0 (19.8)	63.5 (15.6)	63.5 (19.5)	0.788 °
Abduction ipsi	158.1 (36.0)	171.5 (16.5)	167.5 (18.9)	174.5 (13.5)	0.237 †
Forward flexion ipsi	168.3 (17.4)	174.0 (11.7)	174.6 (7.1)	175.0 (7.5)	0.176 †
AROM neck					
Rotation ipsi	53.7 (12.0)	53.0 (14.0)	63.0 (7.9)	63.5 (16.3)	0.008 °**
Rotation contra	55.8 (11.0)	59.0 (11.7)	62.5 (6.7)	62.0 (7.5)	0.029 °*
Flexion	49.7 (12.0)	50.0 (14.5)	56.5 (11.6)	52.5 (15.0)	0.086 °
Extension	53.1 (10.4)	52.0 (18.6)	56.8 (14.1)	59.0 (24.7)	0.362 °
Lateral flexion ipsi	36.0 (12.7)	33.5 (13.5)	36.8 (6.6)	36.5 (7.3)	0.349 †
Lateral flexion contra	36.0 (13.5)	35.5 (12.5)	38.4 (5.5)	38.0 (6.7)	0.468 °
EORTC-QLQ-C30 ¥					
Global Quality of Life	79.4 (14.5)	83.3 (20.8)	82.1 (13.0)	83.3 (22.9)	0.729 †
Physical functioning	88.2 (14.2)	93.3 (23.3)	88.0 (16.4)	93.3 (13.3)	0.964 †
Role functioning	81.4 (28.2)	100.0 (33.3)	85.8 (26.6)	100.0 (29.2)	0.598 †
Emotional functioning	84.3 (20.8)	91.7 (29.2)	82.5 (19.5)	87.5 (31.3)	0.707 †
Cognitive functioning	83.3 (25.0)	100.0 (16.7)	89.2 (9.8)	83.3 (16.7)	0.845 †
Social functioning	89.2 (17.7)	100.0 (16.7)	89.2 (17.3)	100.0 (16.7)	0.916 †
EORTC-QLQ-HN35					
Oral Pain	15.7 (21.6)	0.0 (29.2)	15.0 (17.0)	8.3 (25.0)	0.675 †
Swallowing problems	12.0 (22.0)	0.0 (16.7)	8.7 (12.5)	0.0 (20.8)	0.988 †
Senses problems	10.2 (19.1)	0.0 (8.3)	11.7 (14.4)	8.3 (16.7)	0.426 †
Speech problems	14.2 (23.7)	0.0 (33.3)	13.9 (13.4)	11.1 (22.2)	0.317 †
Trouble with social eating	19.4 (30.4)	0.0 (25.0)	9.2 (11.8)	0.0 (22.9)	0.534 †
Trouble with social contact	6.3 (9.8)	0.0 (13.3)	3.3 (8.2)	0.0 (0.0)	0.313 †
Less sexuality	8.3 (20.0)	0.0 (0.0)	15.8 (22.6)	0.0 (33.3)	0.276 †
Teeth problems	19.3 (15.4)	0.0 (33.3)	11.7 (24.8)	0.0 (25.0)	0.942 †
Trouble with opening mouth	14.8 (23.5)	0.0 (33.3)	13.3 (27.4)	0.0 (25.0)	0.718 †
Dry mouth	33.3 (32.3)	33.3 (66.7)	23.3 (24.4)	33.3 (33.0)	0.409 †
Sticky saliva	12.3 (23.3)	0.0 (33.3)	10.0 (15.7)	0.0 (33.3)	0.965 †
Coughing	16.7 (23.6)	0.0 (33.3)	20.0 (29.4)	0.0 (33.0)	0.874 †
Feeling ill	5.6 (12.8)	0.0 (0.0)	15.0 (22.9)	0.0 (33.0)	0.303 †

¥ The EORTC-QLQ-C30 scores had 1 patient in the END group with missing data. *: *p* < 0.05; **: *p* < 0.01; †: Mann–Whitney U test; °: independent *t*-test. AROM: Active Range Of Motion; END: Elective Neck Dissection; EORTC-QLQ-C30: European Organization for Research and Treatment of Cancer Quality of Life Questionnaire; EORTC-QLQ-H-N35: European Organization for Research and Treatment of Cancer Quality of Life Questionnaire Head & Neck; IQR: interquartile range; NDI: Neck Disability Index; NDII: Neck Dissection Impairment Index; SDQ: Shoulder Disability Questionnaire; SNLB: sentinel lymph node biopsy; SPADI: Shoulder Pain And Disability Index.

## Data Availability

The data used in this study are available upon request.
